# Enhanced diffuse optical tomographic reconstruction using concurrent ultrasound information

**DOI:** 10.1098/rsta.2020.0195

**Published:** 2021-08-23

**Authors:** G. Di Sciacca, L. Di Sieno, A. Farina, P. Lanka, E. Venturini, P. Panizza, A. Dalla Mora, A. Pifferi, P. Taroni, S. R. Arridge

**Affiliations:** ^1^ Department of Computer Science, University College London, London WC1E 6BT, UK; ^2^ Dipartimento di Fisica, Politecnico di Milano, Piazza Leonardo da Vinci, 32 20133 Milano, Italy; ^3^ Consiglio Nazionale delle Ricerche, Istituto di Fotonica e Nanotecnologie, Piazza Leonardo da Vinci, 32 20133 Milano, Italy; ^4^ Breast Imaging Unit, San Raffaele Scientific Hospital, Milano, Italy

**Keywords:** inverse problems, optical, ultrasound

## Abstract

Multimodal imaging is an active branch of research as it has the potential to improve common medical imaging techniques. Diffuse optical tomography (DOT) is an example of a low resolution, functional imaging modality that typically has very low resolution due to the ill-posedness of its underlying inverse problem. Combining the functional information of DOT with a high resolution structural imaging modality has been studied widely. In particular, the combination of DOT with ultrasound (US) could serve as a useful tool for clinicians for the formulation of accurate diagnosis of breast lesions. In this paper, we propose a novel method for US-guided DOT reconstruction using a portable time-domain measurement system. B-mode US imaging is used to retrieve morphological information on the probed tissues by means of a semi-automatical segmentation procedure based on active contour fitting. A two-dimensional to three-dimensional extrapolation procedure, based on the concept of distance transform, is then applied to generate a three-dimensional edge-weighting prior for the regularization of DOT. The reconstruction procedure has been tested on experimental data obtained on specifically designed dual-modality silicon phantoms. Results show a substantial quantification improvement upon the application of the implemented technique.

This article is part of the theme issue ‘Synergistic tomographic image reconstruction: part 2’.

## 1. Introduction

Diffuse optical tomography (DOT) is a medical imaging technique based on the injection and collection of near infrared and visible light in tissue [1]. Recent progress in this technology has seen significant advances in developing miniaturized devices at low cost [2,3]. However, clinical uptake of this modality has been limited by the low resolution, artefacts and high noise in the reconstructed images, which is a consequence of the severe ill-posedness of the inverse problem. Despite these drawbacks, DOT is still an active and growing branch of research worldwide because of its non-invasiveness and cost-effectiveness with respect to other commonly used imaging techniques [4]. Moreover, the spectral nature of DOT admits a spectroscopic and therefore functional analysis of soft tissues that is crucial for clinical diagnosis [5–8]. A leading application for DOT is in breast cancer diagnosis [9]. Nowadays, commonly used imaging protocols for this application are computed tomography (CT), magnetic resonance imaging (MRI) and ultrasound (US) imaging. Of these, US imaging is regarded as the state of the art and the main tool for breast cancer screening. It is an established imaging technique and benefiting from cost-effectiveness, non-invasiveness and a sensitivity close to 100%. The high resolution of US B-mode images leads to clinical diagnosis based on detecting anomalous morphology [10]. Additionally, shear wave elastography (SWE) and colour Doppler ultrasound (CDU) give relevant insight on the presence of collagen and on oxygenation [11]. Nevertheless, the specificity of US imaging is around 80% [12], and, consequently, patients need to go through expensive and often invasive medical exams for a confirmatory diagnosis [13]. Thus, adopting DOT as a suitable complementary technique, e.g. in a multi-modal probe [14], could lead to significant economical and clinical benefits. The interest in multimodality imaging is advancing with the ever increasing progress in systems and reconstruction techniques [15,16]. The idea of combining DOT with a higher resolution, well-posed, structural imaging modality was suggested early in the development of the field [17,18], and the availability of more than one modality in a single probe naturally leads to the question of how to best combine them rather than just treating them separately [19–21]. In particular the combination of DOT with ultrasound (US) has shown promising results, especially in regard to breast cancer diagnosis [18,22–24]. At the same time, the combination of optical and acoustic measurements has led to several *coupled physics imaging* modalities such as photoacoustic tomography (PAT) [25] and ultrasound modulated optical tomography (UMOT) [26]. These methods are so named because the measurement involves the cross-generation of one type of wave from another [27]. Efforts have been spent in designing new dual probes [9,28] and in characterizing tumours also by their reconstructed absorption as a means to better classify them and predict their reactions to therapy [29–32]. In this paper, we describe the study of a combined US-DOT set-up based on the time-domain probe being developed by the SOLUS consortium [33]. We give a short introduction to the main mathematical methods of time-domain DOT as well as on previous applications of US imaging as a prior. After presenting a method for the extraction of relevant information from US B-mode images and their application to generate structural priors for optical reconstruction, we show its applicability on experimental data gathered from specifically developed silicone phantoms.

## 2. Image reconstruction in diffuse optical tomography

DOT is an example of a parameter identification inverse problem where the reconstructed images represent coefficients of a forward model of photon propagation [34,35]. A variety of forward models is possible, of differing complexity, that can account for different aspects of photon behaviour such as directionality, polarization, time-of-flight, etc. [36]. In this paper, we consider a simple model viz. the time-dependent diffusion equation: let Ω be the domain under consideration, with surface ∂Ω; then the photon density (*fluence*, Φ) is given by

2.1 $$\frac{1}{v}\frac{\partial \Phi (\text{r},t)}{\partial t}-\nabla \cdot \kappa \nabla \Phi (\text{r},t)+\mu_{\text{a}}\Phi (\text{r},t)=q(\text{r},t)\;(\text{r},t)\in \Omega \times (0,T],$$

together with appropriate initial and boundary conditions, and where *q* is a source term, *v* is the speed of light, *μ*_a_ is the absorption coefficient and *κ* the diffusion coefficient, which is equal to (3*μ*_s_′)^−1^, where *μ*_s_′ is the reduced scattering coefficient. This model admits explicit Green’s function representation in several geometries; in particular, the three-dimensional infinite space Green’s function

2.2 $$G_{\infty }^{\left(\Phi \right)}({\text{r}}_{2},{\text{r}}_{1},t_{2},t_{1}):=\frac{v}{\sqrt{{\left(4\pi v\kappa (t_{2}-t_{1})\right)}^{3}}}{\text{e}}^{-\frac{|{\text{r}}_{2}-{\text{r}}_{1}{|}^{2}}{4\kappa v(t_{2}-t_{1})}}{\text{e}}^{-\mu_{\text{a}}v(t_{2}-t_{1})},\;t_{2}>t_{1},$$

from which other cases such as half-space and infinite slab geometries follow by the method of images [37]. In the following, we will assume *t*_1_ = 0 unless otherwise stated and use the notation *G*(**r**_2_, **r**_1_, *t*). Note that this form of Green’s function is formally equivalent to solving equation (2.1) with the source *q*(**r**, *t*) to be a delta function in time and space. The response for an experimental system can be modelled by convolving this function with the actual source function with a finite breadth in space and time. We consider *N*_*Q*_ source positions ${\text{q}}_{j}\in \partial \Omega \;(j=1\cdots N_{Q})$ and *M*_*j*_ measurement positions ${\text{r}}_{i}\in \partial \Omega \;(i=1\cdots M_{j})$ for each source *j*. The forward problem is nonlinear and is represented by

2.3 $${\text{F}}_{j,i}:\{\mu_{\text{a}},\kappa \}\to \Phi_{j}({\text{r}}_{i},t)\;\text{r}\text{on}\;\partial \Omega .$$

The objective function, under the hypothesis of a Gaussian noise, is defined as

2.4 $$E(\mu_{\text{a}},\kappa )=\frac{1}{2}\sum_{j=1}^{N_{Q}}\sum_{i=1}^{M_{j}}\sum_{k=1}^{N_{\text{TW}}}\frac{{\left(\int_{T_{k}}^{T_{k+1}}y_{j,i}(t)-{\text{F}}_{j,i}(t,\mu_{\text{a}},\kappa )\text{d}t\right)}^{2}}{\int_{T_{k}}^{T_{k+1}}\sigma_{j,i}^{2}(t)\text{d}t},$$

where *y*_*j*,*i*_(*t*) is the time-resolved data for the *i*th measurement from source *j* with standard deviation *σ*_*j*,*i*_(*t*) and *N*_TW_ is a number of temporal windows adopted for ease of computation.

### (a) Linear inversion method

In this paper, we take a simple linear inversion approach for image reconstruction

2.5 $$y(r,t)=y_{0}(r,t)+y^{\delta }(r,t)≃F(t,\mu_{\text{a, 0}},\kappa_{0})+\;J\left(\begin{array}{c} \delta \mu_{\text{a}}\\ \delta \kappa \end{array}\right),$$

where  J is the Jacobian of **F** obtained for a reference set of optical coefficients *μ*_a_, 0 and *κ*_0_. As discussed in [34], the perturbation *y*^*δ*^ of the boundary measurement *y* due to a perturbation *δμ*_a_(**r**′) and *δκ*(**r**′) in Ω is given by the Born approximation

2.6 $$\begin{array}{rl} y^{\delta }(r,t) & =-\int_{-\infty }^{\infty }\int_{\Omega }\delta \mu_{\text{a}}(r^{\prime })G^{\left(y\right)}(r,r^{\prime },t^{\prime })\Phi (r^{\prime },q,t-t^{\prime })\text{d}r^{\prime }\text{d}t^{\prime }\\ & \;-\int_{-\infty }^{\infty }\int_{\Omega }\delta \kappa (r^{\prime })\nabla^{\prime }G^{\left(y\right)}(r,r^{\prime },t^{\prime })\cdot \nabla^{\prime }\Phi (r^{\prime },q,t-t^{\prime })\text{d}r^{\prime }\text{d}t^{\prime }, \end{array}$$

where *G*^(*y*)^(**r**, **r**′, *t*′) is the Green function for a detector placed at the position r∈∂Ω, due to a delta-like source in $r^{\prime }\in \Omega$ at the time *t*′ and Φ(**r**′, **q**, *t* − *t*′) is the Green function for a detector in **r**′ due to a delta-like source at q∈∂Ω at the time *t* − *t*′. Owing to a reciprocity relation [34,38], it is valid that

2.7 $$G^{\left(y\right)}(r,r^{\prime },t^{\prime })=\Phi (r^{\prime },r,t^{\prime })$$

which allows the computation of *G*^(*y*)^ simply by placing a source at the position of the detector. We observe that equation (2.6) is based on two time convolutions which are typically computed by multiplication in the Fourier domain. Moreover, after this operation, *y*^*δ*^(**r**, *t*) is binned into *N*_TW_ temporal windows to avoid computationally intractable matrices.

The above leads to the representation of the Jacobian  J as

2.8 $$\left(\begin{array}{c} b_{1}\\ b_{2}\\ \vdots \\ b_{N_{Q}} \end{array}\right)=\left(\begin{array}{cc} J_{1,(\mu_{\text{a}})} & J_{1,(\kappa )}\\ J_{2,(\mu_{\text{a}})} & J_{2,(\kappa )}\\ \vdots & \vdots \\ J_{N_{Q},(\mu_{\text{a}})} & J_{N_{Q},(\kappa )} \end{array}\right)\left(\begin{array}{c} \delta \mu_{\text{a}}\\ \delta \kappa \end{array}\right).$$

The blocks are expressed as follows:

2.9 $$b_{j}=\left(\begin{array}{c} y_{j,1}^{\delta }\\ y_{j,2}^{\delta }\\ \vdots \\ y_{j,M_{j}}^{\delta } \end{array}\right),\;J_{j,(\mu_{\text{a}})}=\left(\begin{array}{c} G_{1}*\Phi_{j}\\ G_{2}*\Phi_{j}\\ \vdots \\ G_{M_{j}}*\Phi_{j} \end{array}\right),\;J_{j,(\kappa )}=\left(\begin{array}{c} \nabla G_{1}*\nabla \Phi_{j}\\ \nabla G_{2}*\nabla \Phi_{j}\\ \vdots \\ \nabla G_{M_{j}}*\nabla \Phi_{j} \end{array}\right).$$

where each element is a column vector of length *N*_TW_. Thus, the final resulting Jacobian is a matrix having *N*_*Q*_ × *M*_*j*_ × *N*_TW_ rows and 2*N* columns where *N* is number of voxels in the computational grid.

### (b) Ultrasound imaging and its role as a prior

US B-mode imaging is a well-established technique in breast cancer diagnosis [10,39]. The working principle is the propagation of acoustic waves through human tissues which are described by a characteristic impedance [12]. Assuming the speed of an input acoustic wave to be fixed to the one of water, acoustic pulses are sent through the medium. The reflection phenomena in the tissues cause echoes to be recorded by a receiver. The time delays between the input wave and the returning signals give an indication of the depth at which the reflection occurred. Thus, when a change of impedance along the propagation direction of the wave is measured, a bright spot at a given depth can be drawn. A serial acquisition of such signals over a plane results in an B-mode image. Nowadays, there is also the possibility of acquiring information from three-dimensional portions of human tissues [40]; however, the majority of US probes used in clinical settings are designed to image planes rather than volumes. Over the last decades, medical imaging through multi-modal acquisition has seen a rising interest because of its potential in overcoming the limitations of a single technique approach. The high resolution of US imaging has made it a natural choice to furnish complementary prior information to apply in DOT reconstructions, which are affected by a low space resolution and by uncertainty both in the localization of breast lesions as well as in the evaluation of its components [18]. Image reconstruction in DOT, considered as an inverse problem, is severely ill-posed and calls for the use of *regularization* techniques. An established approach for this is to augment the objective function in equation (2.4) with a *penalty term*, $\tilde{E}=E+\alpha R$, which is designed to control the propagation of noise in the images and/or to enhance desirable features and *α* is a scaling term controlling the effect of the regularization. Regularization techniques are typically based on global descriptors such as the overall magnitude of the reconstructed images and/or image features such as edges and can be expressed in simple mathematical expressions such as zero- and first-order Tikhonov functionals [41]. When considering how to make use of an auxiliary modality such as the US data we are employing here, we need to design the penalty term appropriately. For a general overview of regularization strategies in multimodality imaging see [42] and the references therein. For applications of these methods in US-DOT, strategies differ between those that group pixels to impose a lower dimension in the space being solved in the problem specified in equation (2.8), and those keeping a uniform resolution and imposing an image-based prior. In the dual-mesh approach [43], the prior information on the precise localization of breast inclusions given by the US is used to define a region of interest (ROI) in the computational grid with a finer mesh and a background with larger grid points. The total number of parameters of the inverse problem is thus reduced with a consequent gain of contrast in the reconstructed images. Based on this technique, a combined US-DOT probe with a three-dimensional US array [28] was used to retrieve a three-dimensional ROI. The difficulty in deriving a three-dimensional structure of a lesion from US measurements led to methods with constraints on the ROI search space [14]. Here, an ellipsoidal inclusion is assumed for a first reconstruction based on the dual-mesh approach. A second step is then aimed at refining the retrieved inclusion by perturbing its centre and radii and selecting the most appropriate combination. Mostafa *et al*. [44] proposed to select the ROI of the ultrasound based on the results of a semi-automatic segmentation with a threshold-based method. The segmentation is shown to perform well on US images characterized by high contrast. However, the ROI was selected on the basis of the maximum elongation of the inclusion in the US imaging plane thus losing part of the information recovered in the segmentation. An imaging based prior was applied to combined US-DOT measurements in [45]. Several US B-mode scans were used as a reference image to directly generate a regularization matrix based on its intensity thus avoiding the explicit segmentation of the images. However, US images of breast lesions are characterized by high variability and artefacts such as shadowing. This poses some challenges to an extensive use of the method. Moreover, arrays of US images might not available during clinical exams. In this paper, we make use of a semi-automatic segmentation of the target region (i.e. a breast lesion) and derive a weighted edge penalty given by

2.10 $$R(x;U)=\int_{\Omega }\gamma (U)|\nabla x{|}^{2}\text{d}r,$$

where *x* represents *μ*_a_ or *κ* and *γ*(*U*) ∈ [0, 1] is a function that tends to zero at the locations of identifiable edges in *U* and tends to 1 in regions that do not contain significant structural information. We remark that the form of regularization in equation (2.10) leads to an interpretation of its derivative as a form of *anisotropic filtering* [46,47]. To impose the (approximate) segmentation, we choose

2.11 $$\gamma =\exp\left[-\frac{|\nabla \chi |}{\beta }\right],$$

where *χ* is derived from a two-dimensional US image $U_{P}$ via a process of two-dimensional curve fitting and extrapolation to three dimensions and *β* is a threshold parameter; here P specifies the plane in which the US images is taken. The detailed extraction procedure to derive *χ* from $U_{P}$ is described in §3. Combining equations (2.4), (2.5) and (2.10) leads to the inverse problem as the minimization of the following objective problem:

2.12 $$\left\|\left(\begin{array}{c} \;S\;J\\ \alpha^{1/2}\;L \end{array}\right)\left(\begin{array}{c} \delta \mu_{\text{a}}\\ \delta \kappa \end{array}\right)-\left(\begin{array}{c} \;Sb\\ 0 \end{array}\right)\right\|{}^{2}\to \min,$$

where  S is a diagonal matrix of the inverse standard deviations of each measurement 1/*σ*_*j*,*i*_ and  L is a matrix decomposition chosen so that $\left\langle f,{\;L}^{\text{T}}\;L\;f\right\rangle \equiv R(f)$ for *f* ∈ [*δμ*_a_, *δκ*]. We use LSQR to solve equation (2.12).

### (c) Phantoms

**Figure 1.**
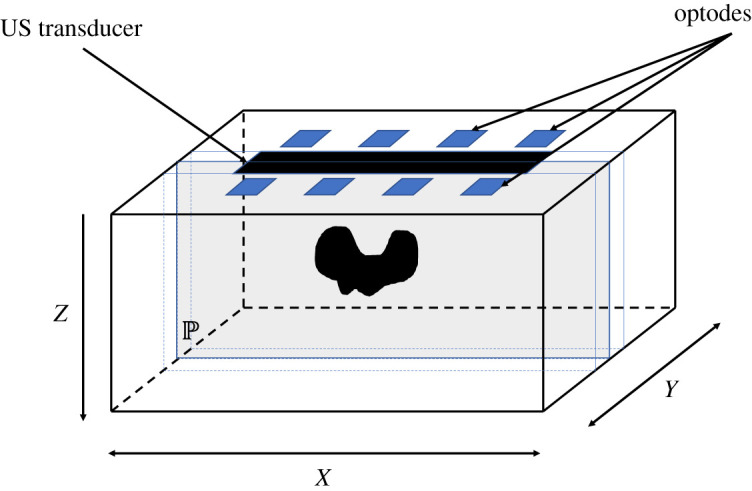
Schematic of measurements geometry. Detectors and sources are aligned in two rows parallel to the direction of the US scan. The plane of US imaging P is set to be at *y* = 0. The plane *z* = 0 identifies the surface of the domain. (Online version in colour.)

Joint US-DOT heterogeneous phantoms were specifically developed by the SOLUS consortium for this study and for future experimental validation of the multimodal probe. Their detailed description can be found in [48]. Solid materials were preferred to obtain stable and durable phantoms. In particular, an echogenic contrast for US investigations between bulk and perturbation phantoms was obtained thanks to the combination of two different silicones: the Ecoflex 00-30 (Smooth-On, Inc. PA, USA) silicone rubber (used for the bulk, i.e. a rectangular parallelepiped featuring a 100 × 120 mm^2^ surface and a 40 mm thickness) and the Sylgard S184 (Dow Corning Corp. CA, USA) silicone elastomer (used for perturbations, i.e.cylinders with volumes of 1 cm^3^—11 mm diameter, 10 mm height—or 6 cm^3^—22 mm diameter, 15 mm height). Since echogenicity in Sylgard S184 is lower than in Ecoflex 00-30, their combination allowed us to simulate anechoic lesions inside echogenic tissues. The bulk phantom had on one surface two cylindrical cavities (volume of 1 cm^3^ and 6 cm^3^) where perturbations of equivalent volume can be inserted. Three slices (with thicknesses of 5, 15 or 25 mm) made of the same material as the bulk can be used to cover perturbations so as to simulate different depths of the lesion inside the tissue. Both bulk and perturbation phantoms were fabricated with different optical properties by adding different calibrated (at 690 nm wavelength) quantities of titanium (IV) oxide powder (Sigma Aldrich, USA) as a scattering element and toner powder (Infotec, Toner Black 46/l) as absorber, thus permitting an almost flat absorption coefficient over a broad spectral range. It is worth noting that the recipe was validated demonstrating the independence between acoustic properties and the concentration of absorbers and scatterers (in the concentrations of interest for this study) [48]. Here, a bulk phantom with (nominally) *μ*_s_′ = 1 mm^−1^ and *μ*_a_ = 0.01 mm^−1^ was used, with a top slice of 5 mm thickness (featuring same optical properties as the bulk) covering a 1 cm^3^ perturbation with (nominally) *μ*_s_′ = 1 mm^−1^ and *μ*_a_ = 0.005, 0.01, 0.02, 0.04 or 0.06 mm^−1^. For the sake of the present study, US measurements were taken using an SL18-5 US probe connected to an Aixplorer v.11 system (Supersonic Imagine S.A., France) operated with customized settings (optimized for imaging into silicone phantoms, e.g. transmit and receive demodulation frequency = 5 MHz, voltage = 72 V, 2 half cycles, speed of sound set to 980 m s^−1^). DOT measurements presented here were taken using a time-resolved diffuse spectroscopy laboratory research prototype [49] in a scanning fashion. The instrumental response function, taking into account the finite width of the light source, and other possible broadening factors, such as fibre temporal dispersion, detector response, etc., is used in the generation of the Jacobian by convolution with equation (2.6). Both source and detection fibres were manually switched between 8 different locations (holes into a black polyvinyl chloride plate placed on top of the phantom), thus resulting into a single DOT acquisition composed by 64 source-detector pairs. The eight locations were chosen so as to replicate the SOLUS probe geometry [11] (characterized by 8 DOT acquisition points around the US transducer). In detail, the acquisition geometry was composed by two lines of four measurement points each. Measurement points on the same line are at an intermediate distance of 12 mm, while the two lines are separated by 20 mm. A schematic of the acquisition geometry can be found in figure 1. Each assembled phantom was probed over eight wavelengths of 635, 670, 830, 915, 940, 980, 1030 and 1065 nm. The measurements were performed in time-domain and normalized by area.

## 3. Generating a structural prior

### (a) Segmentation using snakes

In order to identify semi-automatically the region where an inclusion is present in a Ultrasound B-mode image $U_{P}$, a Snake-based method was implemented [50]: given some user-defined control points $\left\{{p^{\left(0\right)}}_{i};\;i=1\ldots N\right\}$ close to the edges of the inclusion, an active contour fitting procedure finds an optimal set {p*_i_; *i* = 1…*N*} whose cubic spline interpolation $C_{P}(s)$ approximates the border of the lesion. An appropriate energy term E was defined as the sum of two contributions

3.1 $$E(C_{P})=\int_{0}^{1}E_{\text{int}}(C_{P}(s))+E_{\text{im},\sigma }(C_{P}(s))\text{d}s,$$

and minimized. The first contribution *E*_int_ is related to the internal energy of $C_{P}(s)$ is given by

3.2 $$E_{\text{int}}=\frac{1}{2}\left(a(s){\left|\frac{\text{d}}{\text{d}s}C_{P}(s)\right|}^{2}+b(s){\left|\frac{{\text{d}}^{2}}{\text{d}s^{2}}C_{P}(s)\right|}^{2}\right),$$

where the choice of *a* and *b* affects the appearance of $C_{P}(s)$ such as the presence of corners. The term *E*_im,*σ*_ in the second contribution of equation (3.1) takes into account the characteristic features of $U_{P}$. The characteristics of US B-mode images allow to look for inclusions’ borders where a smoothed version of the Laplacian

3.3 $$U_{P_{\sigma }}^{\text{Lap}}=G_{\sigma }(r)*\nabla^{2}U_{P}(r)$$

is small enough, with $G_{\sigma }(r)=A{\text{e}}^{-(|r{|}^{2}/2\sigma^{2})}$. In these settings, a characteristic function is defined so that

3.4 $${\chi_{P}}_{U_{P},\sigma ,10\text{\%}}=\left\{ \begin{array}{ll} 1 & \text{if}\;U_{P_{\sigma }}^{\text{Lap}}<P_{10\text{\%}}(|U_{P_{\sigma }}^{\text{Lap}}|)\\ 0 & \text{otherwise} \end{array}\right.;\;E_{\text{im},\sigma }=\text{DT}{\left({\chi_{P}}_{U_{P},10\text{\%}}\right)}^{2},$$

where *P*_10%_ represents the percentile value of the first 10% pixels with lowest values and the threshold of 10% was chosen empirically. The application of the square of the distance transform (DT) (DT) [51,52] on ${\chi_{P}}_{U,10\text{\%}}$ furnishes a potential which is minimum along the relevant features of the lesion and quadratically increasing with the distance from them. This is valid defining DT on the Euclidean distance from the *zero* pixels of *U*. A faster and more robust convergence, has been obtained by using two further expediences. With a small change in the procedure, the user is asked to select the points close to the borders of the inclusion and *on its internal side*. An additional term can then be added to *E*_im_ so that

3.5 $$E_{\text{im},\sigma }=\text{DT}{\left({\chi_{P}}_{U,10\text{\%}}\right)}^{2}+\text{DT}{\left({\chi_{P}}^{\left(0\right)}\right)}^{2},$$

where $\text{DT}{\left({\chi_{P}}^{\left(0\right)}\right)}^{2}$ acts as a repulsive term from the user-selected area, thus avoiding local minima due to the most internal structures of the lesion. A snake segmentation is then found by minimizing equation (3.1) with respect to the control points, i.e.

3.6 $$\{p_{i}^{*}\}=\underset{\left\{p_{i}\right\}}{\text{argmin}}\;E(\text{Spline}(\{p_{i}\}))\;i=1\ldots N.$$

**Figure 2.**
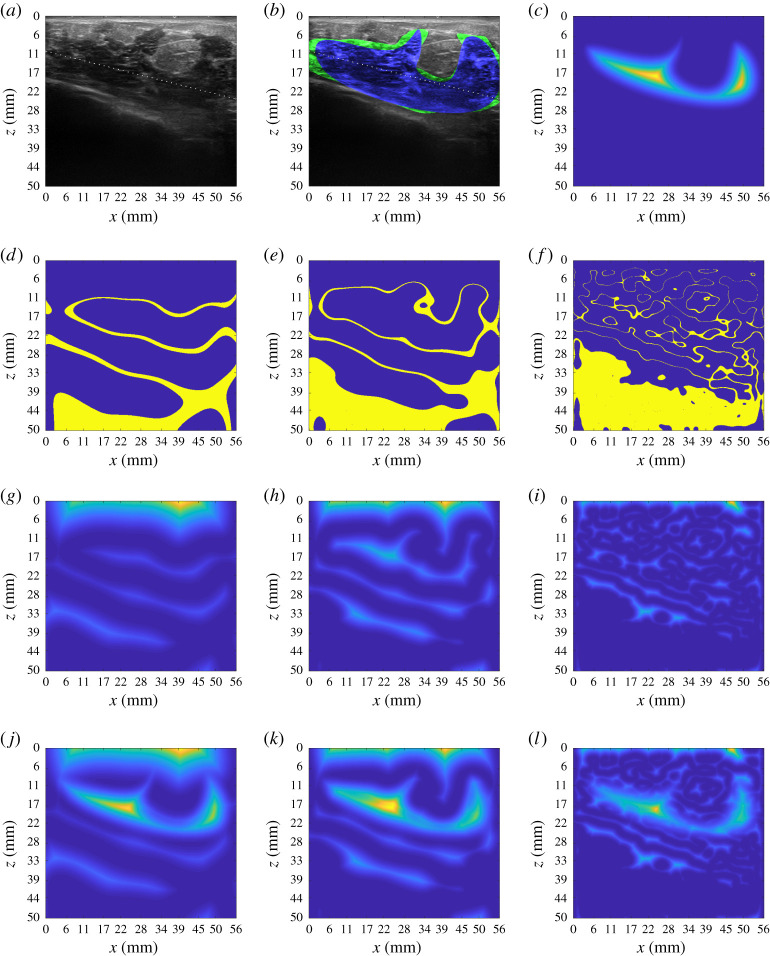
Example of application of semi-automatic segmentation as in Algorithm 1 on US image of breast lesion, courtesy of Ospedale San Raffaele, Milano, Italy. The characteristic length for the multiscale snake was found to be $\hat{r}=75\;\text{pixels}$. The initial segmentation was obtained with the spline interpolation of 10 points. From figure 2*d* to *f* , a subset of $U_{P_{\sigma }}^{\text{Lap}}$ is displayed. The features identified in $U_{P}$ are shown to be refined as *σ* decreases. However, also internal structures are highlighted in the process. The same behaviour, with a clear focus on the local minima of the potentials, can be observed from figure 2*g*,*i* which shows the respective $\text{DT}{\left(U_{P_{\sigma }}^{\text{Lap}}\right)}^{2}$. The addition of $\text{DT}{\left({\chi_{P}}^{0}\right)}^{2}$ in figure 2*c* acts then as a repulsive term towards the local minima inside the initial segmentation. From figure 2*j* to *l*, the full function *E*_im_ is shown. Figure 2*b* shows in detail the improvement brought in the segmentation from our snake-based method. (*a*) Original Image $U_{P}$, (*b*) blue: ${\chi_{P}}^{\left(0\right)}$, green: final segmentation $\chi_{P}$, (*c*) $\text{DT}{\left({\chi_{P}}^{\left(0\right)}\right)}^{2}$, (*d*) ${\chi_{P}}_{U,\sigma_{1},10\text{\%}}$, (*e*) ${\chi_{P}}_{U,\sigma_{8},10\text{\%}}$, (*f* ) ${\chi_{P}}_{U,\sigma_{15},10\text{\%}}$, (*g*) $\text{DT}{\left({\chi_{P}}_{U,\sigma_{15},10\text{\%}}\right)}^{2}$, (*h*) $\text{DT}{\left({\chi_{P}}_{U,\sigma_{8},10\text{\%}}\right)}^{2}$, (*i*) $\text{DT}{\left({\chi_{P}}_{U,\sigma_{15},10\text{\%}}\right)}^{2}$, (*j*) $E_{\text{im},\sigma_{1}}$, (*k*) $E_{\text{im},\sigma_{8}}$ and (*l*) $E_{\text{im},\sigma_{15}}$. (Online version in colour.)

**Figure 3.**
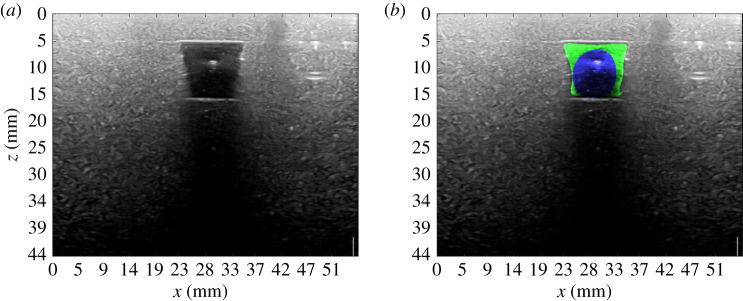
Applicationof semi-automatic segmentation on US images taken on bimodal phantoms, courtesy of Supersonic Image, Aix-en-Provence, France. (*a*) Original image, (*b*) blue: $\chi_{P}^{\left(0\right)}$, green: Final segmentation $\chi_{P}$. (Online version in colour.)

A solution suggested in [53] introduces a parallel minimization in scale-space [54] so that the minimization is not totally affected by the choice of a single *σ*. In this scenario, a set of {*σ*_*k*_; *k* = 1…*N*_*σ*_} is chosen and minimization of E is operated for each predefined smoothing from the largest *σ*_max_ to the smallest one *σ*_min_. A characteristic length $\hat{r}=||\text{DT}(\chi^{\left(0\right)})|{|}_{\infty }$ was defined. The set {*σ*_*k*_; *k* = 1…*N*_*σ*_} of *N*_*σ*_ evenly spaced *σ* was defined between $\sigma_{\text{max}}=0.7\hat{r}$ and $\sigma_{\text{min}}=0.15\hat{r}$. The final process is given in Algorithm 1. The internal energy parameters *a* = 0 and *b* = 1 were chosen.  *N*_*σ*_ was set to be 15. In figure 2, a segmentation example obtained with described method is shown. In figure 3, we show the result of the application of the segmentation procedure to an image *U* taken on one of the bi-modal phantoms developed at Politecnico di Milano.


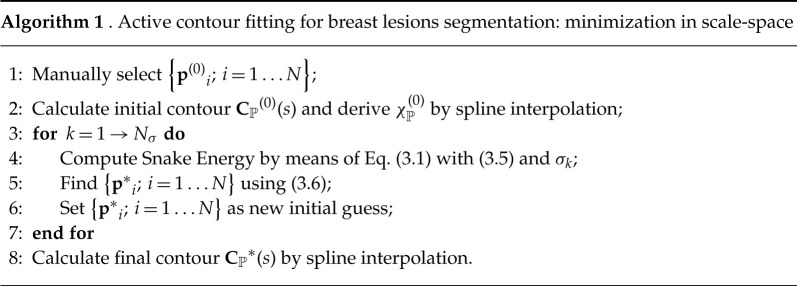


### (b) Extrapolation to three dimensions

In order to apply a structural prior as defined in equations (2.10) and (2.11), a three-dimensional characteristic volume *χ* needs to be extrapolated from the segmentation $\chi_{P}$ of $U_{P}$. For this reason, an operator *T* is defined such that

3.7 $$\chi =T\chi_{P}.$$

Finding a form for *T* is not possible without some strong and mostly qualitative assumptions. Here, we present a possible implementation which, as for the snake implementation, makes use of the concept of the DT. The basic idea is to assume that the clinician guiding the US acquisition has selected the slice with the maximal cross-sectional area of the lesion and that the out-of-plane extension of the lesion diminishes smoothly. In order to implement such conditions, a height function *y*_ext_(*x*, *z*) was defined over the plane {(x,z)∈P}. This is designed to control the extension of the inclusion out of the US imaging plane P by extrapolating a smooth shape. Thus:

3.8 $$\begin{array}{rl} y_{\text{ext}}(x,z) & =c\sqrt{2\;\text{DT}(\chi_{P})(x,z)||\text{DT}(\chi_{P})|{|}_{\infty }-\text{DT}{\left(\chi_{P}\right)}^{2}}\\ \text{with}\;\\ c & =\sqrt{\frac{A}{\pi }}||\text{DT}(\chi_{P})|{|}_{\infty } \end{array}$$

where *A* is the area of the inclusion $\chi_{P}$ so that *c*, in the absence of statistical insight on the distribution of the lesions’ shape, ensures that the out-of-plane extrusion is never bigger than the radius of the sphere whose middle section has the same area of the inclusion portrayed in $\chi_{P}$. The extrusion operator will finally read as

3.9 $$\chi (x,y,z)=T^{\text{DT}}\chi_{P}(x,z)=\left\{ \begin{array}{ll} 1 & y^{2}<y_{\text{ext}}^{2}(x,z)\\ 0 & \text{elsewhere}. \end{array}\right.$$

**Figure 4.**
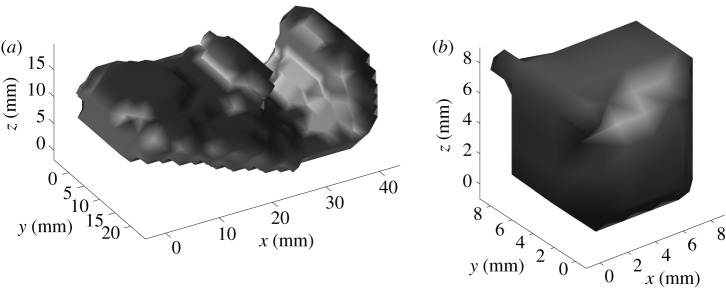
Three-dimensional rendering of the result of the extrapolation performed as described in §3b on $\chi_{P}$ of figure 2 (*a*) and of figure 3 (*b*). (*a*) Extrapolated inclusion *χ* from $\chi_{P}$ shown in figure 2*b*. (*b*) Extrapolated inclusion *χ* from $\chi_{P}$ shown in figure 3. The volume of $\chi_{P}$ is 736 mm^3^. The experimental cylindrical inclusion had volume of approximately 950 mm^3^.

An example of a shape retrieved upon the application of *T*^DT^ on the final segmentation in figure 2*b*, can be seen in figure 4*a*. Results of the final extrapolation procedure on the segmentation shown in figure 3 are shown in figure 4*b*. A validation for *T*^DT^ proves to be complex. An assessment can be made on its effect as a prior in optical reconstructions.

## 4. Results

A validation of the optical reconstruction on phantoms was performed by experimentally reproducing the measurements geometry that is designed for the SOLUS project [11] by making use of specifically designed phantoms described in 2.c. A reference measurement $y_{\text{ref}}^{\left(0\right)}(r,t)$ was obtained on the sole bulk, so that the linearization in equation (2.5) reads as

4.1 $$y(r,t)≃y_{\text{ref}}^{\left(0\right)}(r,t)+\;J\left(\begin{array}{c} \delta \mu_{\text{a}}\\ \delta \kappa \end{array}\right).$$

**Figure 5.**
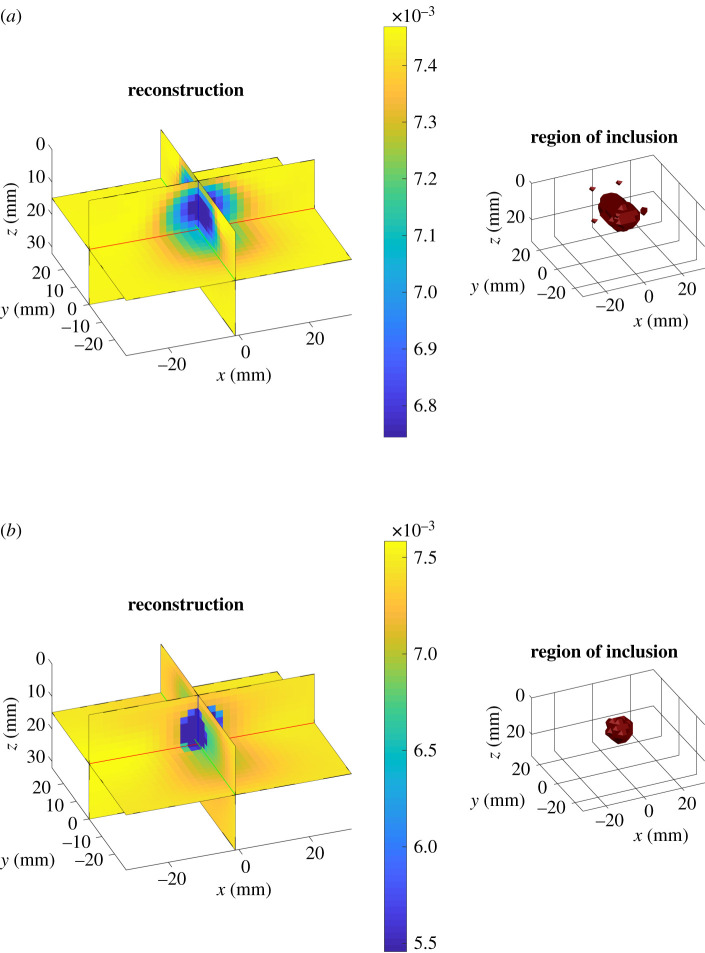
Results for *λ* = 830 nm. *μ*_a_, bulk^true^ = 0.0075 mm^−1^, $\mu_{\text{a},\text{in}}^{\text{true}}=0.0037\;{\text{mm}}^{-1}$. (*a*) Left: absorption reconstruction (mm^−1^) obtained with zeroth-order Tikhonov regularization. Right: volume of the inclusion retrieved with DOT. (*b*) Left: absorption reconstruction (mm^−1^) with edge-weighting prior obtained as described in §3. Right: volume of the inclusion retrieved with DOT. (Online version in colour.)

**Figure 6.**
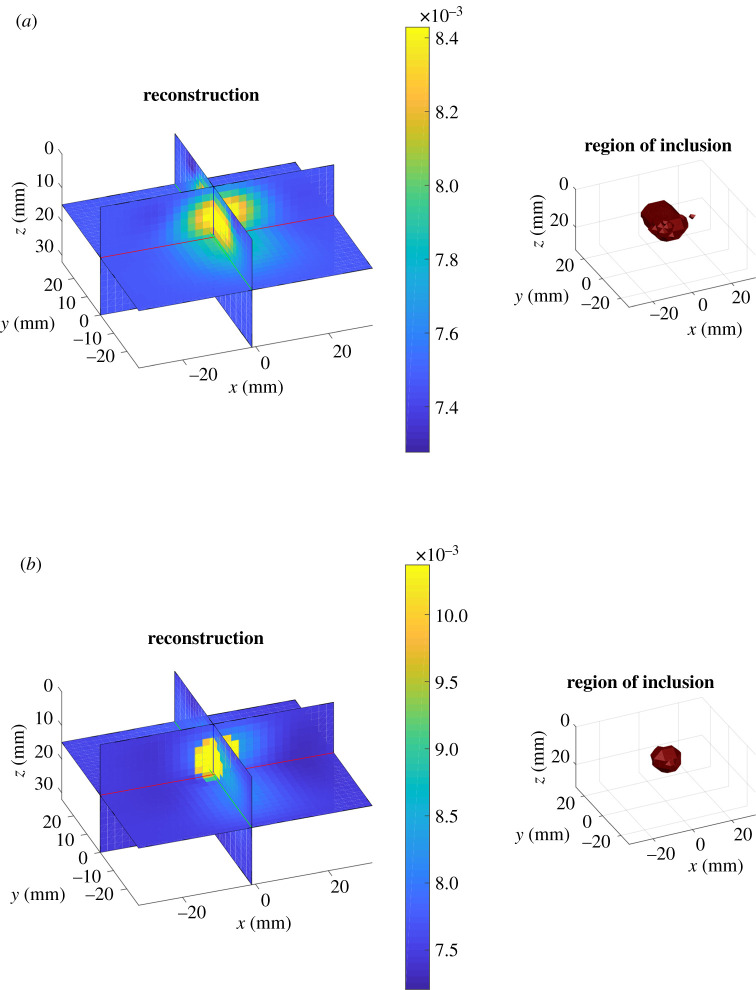
Results. *μ*_a_, bulk^true^ = 0.0075 mm^−1^, $\mu_{a,in}^{\text{true}}=0.0285\;{\text{mm}}^{-1}$. (*a*) Left: absorption reconstruction (mm^−1^) with zeroth order Tikhonov regularization. Right: volume of the inclusion retrieved with DOT. (*b*) Left: absorption reconstruction (mm^−1^) with edge-weighting prior obtained as described in §3. Right: volume of the inclusion retrieved with DOT. (Online version in colour.)

The analytical model was chosen for linearization. Supposing the optical coefficients of the bulk to be known, the Jacobian was built following the discussion in §3a. The reference values of the homogeneous optical coefficients *κ*_0_ and *μ*_a,0_ were assumed to be equal to those in the bulk. The experimental time point spread functions were sampled with a total *N*_TW_ = 20 equally spaced time windows in a manually selected temporal ROI. The reconstruction domain Ω was a cuboid of 64 mm × 58 mm × 32 mm, discretized with cubic voxels of side 2 mm. We compared results with regularization using (i) zeroth-order Tikhonov prior, (ii) edge-weighted prior given by equation (2.10) with the recovered shape using the method of §3. In figure 5 and figure 6, two examples of absorption reconstruction with zeroth-order Tikhonov and with edge-weighting prior are shown. In table 1, more detailed results are given for the examples displayed. It can be seen that the proposed method improves quantification of the optical properties of the inclusion with respect to zeroth-order Tikhonov regularization. This statement holds true also when considering the integral value of the reconstructed *δμ*_a_, in over the region of the inclusion. However, in the case of figure 6 an important discrepancy may be observed between ground truth and reconstructions. This can be partly explained by the inadequacy of the Born approximation in describing situations characterized by high optical contrasts. Also the localization of the inclusion, especially with regard to its elongation, is enhanced by the use of the proposed edge-weighting prior. We also tested using the edge-weighted prior and the exact three-dimensional shape (figures not shown); no particular quantification improvements were found with the exact shape, suggesting that the approximate shape-recovery method is adequate for providing quantitative estimates of the optical properties.

**Table 1.** Table of results for the examples shown in figures 5 and 6. Comparison, for ground truth, reconstructed absorption with zeroth-order Tikhonov regularization and edge-weighting prior, the mean value of the absorption inside the inclusion, the integral of the reconstructed *δμ*_a_, in in the region of the inclusion, the centre of mass of the inclusion along *x*, *y* and *z*, the displacement *d* between the centre of mass of the ground truth and of the reconstructed inclusions, its maximum elongations Δ*x*, Δ*y* and Δ*z* in the computational grid.

	Fig.	*δμ*_a_, in( × 10^−3^ mm^−1^)	$\int_{\omega }\delta \mu_{\text{a, in }}\text{d}r$ (mmˆ2)	*x* (mm)	*y* (mm)	*z* (mm)	*d* (mm)	Δ*x* (mm)	Δ*y* (mm)	Δ*z* (mm)
true	5	-3.8	-3.97	0	0	10	0	12	10	12
	6	21.04	22.2	0	0	10	0	12	10	12
Tikh.	5	-0.6	-1.5	-0.3	-1.3	8.3	2.2	14	24	12
	6	0.84	2.22	-1.2	-1.6	8.6	2.4	16	26	10
R(x;χ)	5	-3.38	-2.81	0.5	-0.6	10.1	0.8	12	12	12
	6	4.87	4.01	0.2	-0.6	9.5	0.8	12	12	12

## 5. Discussion

For a systematic analysis of the results, each reconstructed inclusion was assumed to be localized in the region *ω* defined as

5.1 $$\omega :=\left\{r\;\text{s.t.}\;\left|\frac{\mu_{\text{a}}^{\text{recon}}(r)-\text{median}[\mu_{\text{a}}^{\text{recon}}(r)]}{\text{std}[\mu_{\text{a}}^{\text{recon}}(r)]}\right|>4\right\}.$$

**Figure 7.**
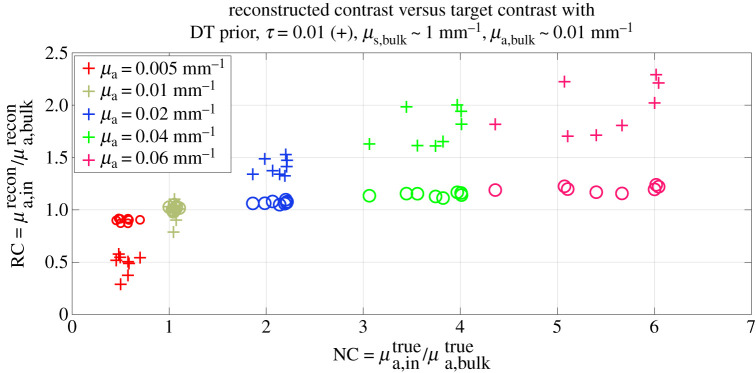
Plot of reconstructed value of the inclusions over the nominal values of the contrast. Crosses indicate the reconstructed values of the inclusion with edge-regularizing prior based on *T*^DT^. Circles are the contrast reconstructed with zeroth-order Tikhonov regularization. (Online version in colour.)

The representative absorption value in the inclusion was then set to be $\mu_{\text{a, in}}^{\text{recon}}=\text{mean}[\mu_{\text{a}}^{\text{recon}}(\omega )]$ while the reconstructed value in the bulk $\mu_{\text{a, bulk}}^{\text{recon}}=\text{mean}[\mu_{\text{a}}^{\text{recon}}(\Omega \setminus \omega )]$ . From the right-hand side of figures 5 and 6, it can be seen that localization of the inclusion is improved with the application our structural prior, as expected. In figure 7, an overview of the quality of the reconstructions with and without DT-based structural prior is given. On the abscissa, the set of the absorption nominal contrast of the probed phantoms as $\text{NC}=\mu_{\text{a, in}}^{\text{true}}/\mu_{\text{a, bulk}}^{\text{true}}$ for all wavelengths is displayed, while the ordinates show the mean reconstructed contrast RC = *μ*_a_, in^recon^/*μ*_a_, bulk^recon^ in the case of the application of a zeroth-order Tikhonov regularization (circles) and of the structural prior (crosses). An optimal reconstruction would see the scatter points lying along the bisector of the axes’ origin. While able to reconstruct the sign of the perturbation correctly, Tikhonov regularization is far from ideal for what regards its intensity. A sensible improvement can be observed when applying a structural prior as the one described in §3.

## 6. Conclusion

We introduced a method for the application of structural priors coming from US imaging to DOT. A semi-automatic segmentation procedure was shown to be able to extract the region of an inclusion from an ultrasound image. A further step based on the concept of DT is then applied to the retrieved segmentation to extrapolate a three-dimensional shape. Such procedure is not expected to accurately represent any three-dimensional lesion, but its use allows to regularize the problem of DOT, with benefits for localization and quantification consequently. The method was assessed by means of experimental data taken of specifically designed phantoms. The application of an edge-prior obtained by means of the devised strategy shows to improve the quantification of DOT reconstructions with respect to plain global regularizations such as zeroth order Tikhonov. Finally, it is shown that quantification is improved systematically for a wide range of optical contrasts between bulk and inclusion.

## Data accessibility

The data and the software used to produce the results shown can be found at: https://github.com/andreafarina/SOLUS/tree/master/example/example_200202_series. Information on how to run the reconstructions are given in a README file.
